# Supplementary material for: Tead1a Initiates Transcriptional Priming Through the TEAD1a/YAP‐Notch1‐Spi1/Cebpα Axis to Promote Neutrophil Fate
Source: Adv Sci (Weinh). 2025 Aug 28;12(41):e05441. doi: 10.1002/advs.202505441 (PMC12591127; doi:10.1002/advs.202505441)
Supplement: Supplementary file 4 — Supplementary Dataset [file ADVS-12-e05441-s004.zip › 3-4.zip!/3-4/02-4.cluster/cluster10/Enrich/ego_all.pdf]

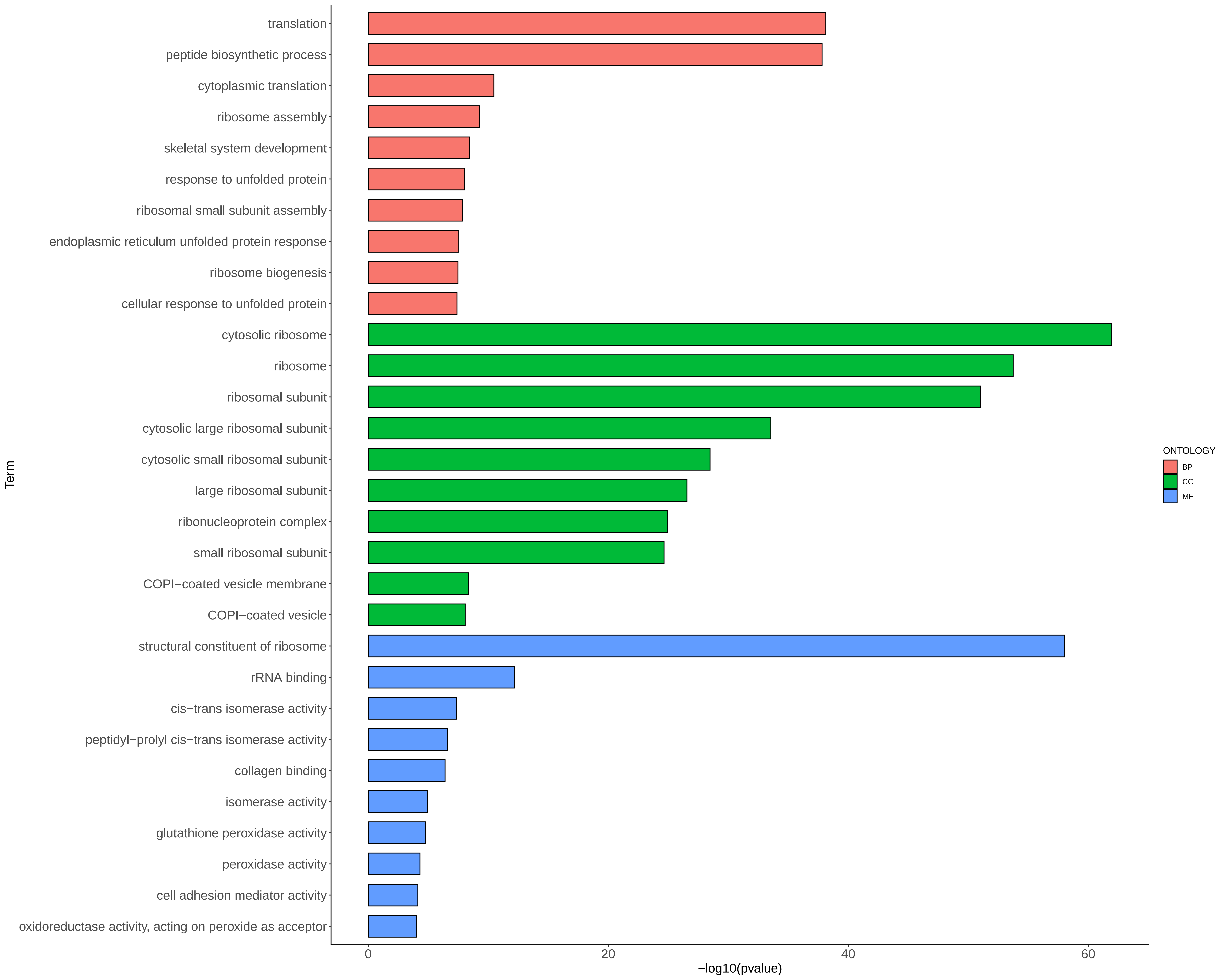
translation
peptide biosynthetic process
cytoplasmic translation
ribosome assembly
skeletal system development
response to unfolded protein
ribosomal small subunit assembly
endoplasmic reticulum unfolded protein response
ribosome biogenesis
cellular response to unfolded protein
cytosolic ribosome
ribosome
ribosomal subunit
cytosolic large ribosomal subunit
cytosolic small ribosomal subunit
large ribosomal subunit
ribonucleoprotein complex
small ribosomal subunit
COPI−coated vesicle membrane
COPI−coated vesicle
structural constituent of ribosome
rRNA binding
cis−trans isomerase activity
peptidyl−prolyl cis−trans isomerase activity
collagen binding
isomerase activity
glutathione peroxidase activity
peroxidase activity
cell adhesion mediator activity
oxidoreductase activity, acting on peroxide as acceptor
Term
0
20
40
60
−log10(pvalue)
ONTOLOGY
BP
CC
MF
